# Intraperitoneal Solitary Fibrous Tumor

**DOI:** 10.1155/2014/906510

**Published:** 2014-09-07

**Authors:** Youssef Benabdejlil, Jaouad Kouach, Abdellah Babahabib, Moulay Elmehdi Elhassani, Issam Rharassi, Adil Boudhas, Hicham Bakkali, Mohammed Elmarjany, Driss Moussaoui, Mohamed Dehayni

**Affiliations:** ^1^Department of Gynecology-Obstetric, Military Training Hospital Med V, Rabat, Morocco; ^2^Faculty of Medicine and Pharmacy, University Mohammed V Souissi, Rabat, Morocco; ^3^Department of Pathological Anatomy, Military Training Hospital Med V, Rabat, Morocco; ^4^Department of Anesthesiology and Critical Care, Military Training Hospital Med V, Rabat, Morocco; ^5^Department of Radiotherapy, Military Training Hospital Med V, Rabat, Morocco

## Abstract

Solitary fibrous tumors of the pelvis are rare. We report the case of a 32-years-old patient who presented with abdominopelvic mass. The imaging studies showed a right adnexal mass of more than 10 cm. Exploratory laparotomy revealed a 20 cm mass at the Douglas pouch which was adhered to the posterior wall of the uterus. Complete resection of the mass was performed. Histological analysis showed a spindle cell undifferentiated tumor whose morphological and immunohistochemical profile are consistent with solitary fibrous tumor. It is important to know that although these tumors are rare, their evolution can be pejorative. Therefore, long-term followup should be recommended.

## 1. Introduction

Solitary fibrous tumors (SFTs) are rare mesenchymal tumors which can be benign or malignant. They occur most often in the pleura, but have also been described in many extrapleural locations. Pelvic localization is very rare.

We first report a case of solitary fibrous tumor revealed by abdominopelvic mass in a 32-year-old woman, and then we describe the clinical, radiological, morphological, and immunohistochemical characteristics of this condition.

## 2. Observation

Mrs. BY, 32-year-old woman, with no medical and family history, gravida 2 para 2, with regular menstrual cycles, using intrauterine contraceptive device (IUD) for three years, was referred by her general practitioner for abdominopelvic mass associated with pelvic pain lasting for one month.

Physical examination showed a 20 cm abdominopelvic mass lateralized to the right, which is regular and very mobile. No lymph nodes were palpated. The pelvic exam found a right adnexal mass that appears to be independent of the uterus. The rest of the physical examination was normal.

**Figure 1:**
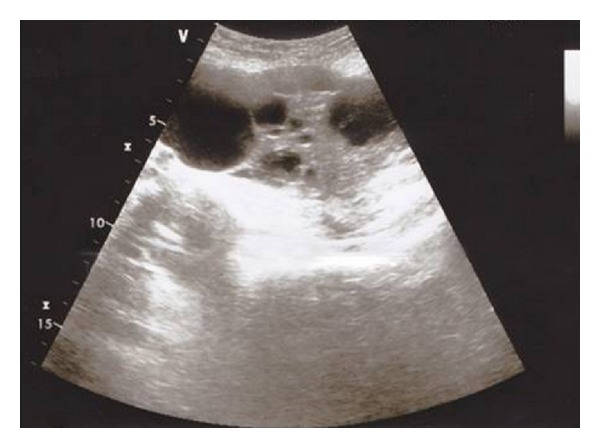
Endovaginal pelvic ultrasound: axial view: Adnexal mass measuring 20 cm, which is heterogeneous, with tissular and cystic components and without intracystic or extracystic exophytic vegetations and without peritoneal fluid.

Pelvic ultrasound with abdominal and endovaginal transducers depicted a right adnexal mass measuring 20 cm, which is heterogeneous, with tissular and cystic components and without intracystic or extracystic exophytic vegetations and without peritoneal fluid. The peritoneal space was free of other abnormalities (Figure 1). The determination of tumor marker CA125 was normal.

**Figure 2:**
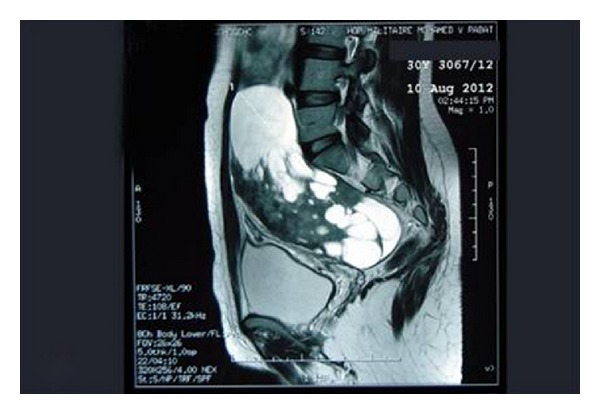
Pelvic MRI: Sagittal T2-weighted image, showing an intraperitoneal tumor with tissular and cystic signal. The tumor is separated from the uterus. The cystic areas appear hyperintense on T2-WI. And the tissular parts present heterogeneous signal on T2 sequence with hypointense areas corresponding to fibrotic tissue. The solid component of the tumor enhances strongly and heterogeneously after gadolinium injection.

**Figure 3:**
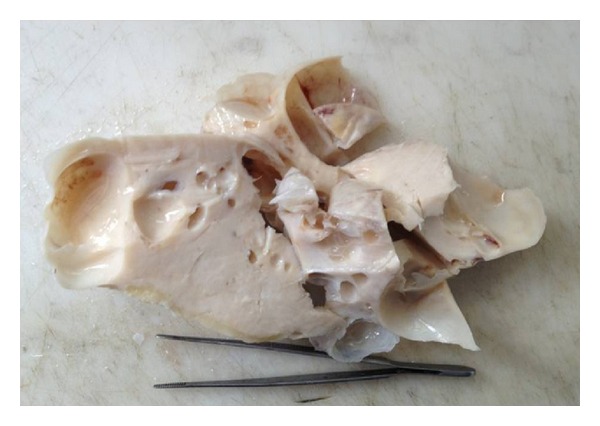
Macroscopic appearance of the tumor: mass measuring 25/22 cm with a smooth and translucent external appearance. On section, the mass is midcystic and midsolid.

**Figure 4:**
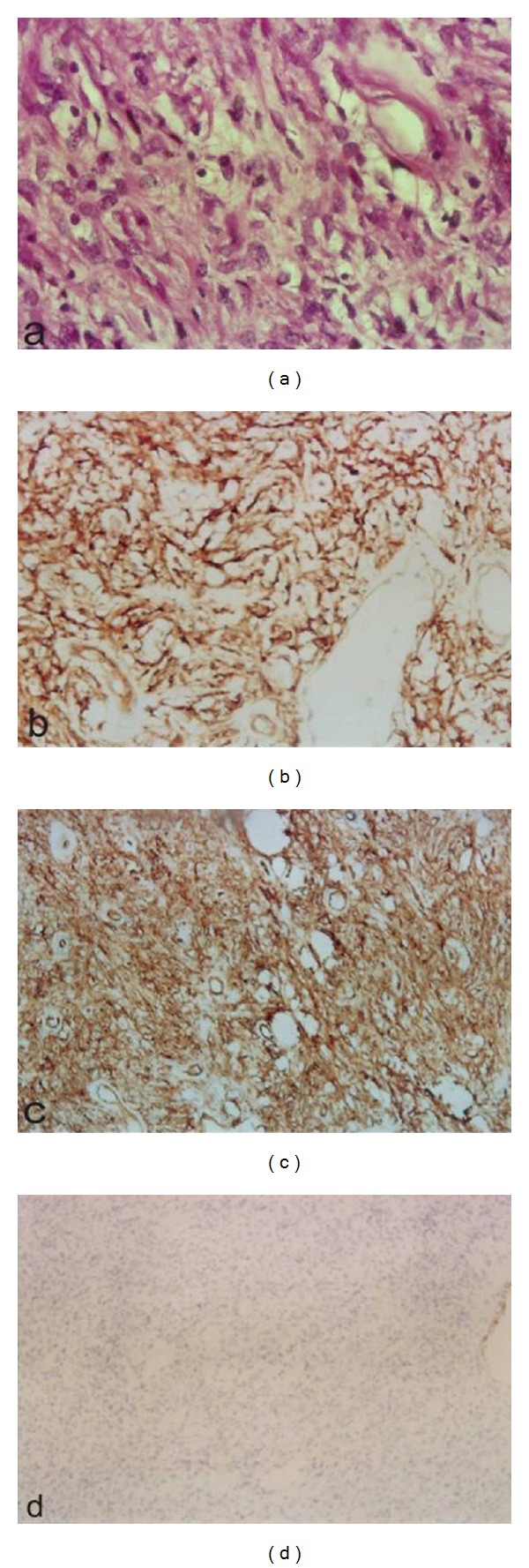
(a) Standard staining HE GX40: slight tumor proliferation with fusocellular cytonuclear atypies. Immunohistochemistry: CD99 antibody positivity (b), anti-CD34 (c), and Ki67 < 1% (d).

Pelvic MRI showed the presence of an adnexal mass, measuring 25/20 cm, which seemed to grow at the expense of the right ovary. The signal was isointense on T1-weighted imaging (WI), with a heterogeneous appearance with alternating hyper- and hypointense zones on T2-WI, as well as a heterogeneous enhancement after gadolinium injection. This mass had a mixed solid and cystic composition with enhancement of the solid parts. The cystic component contains multiple cysts with septations measuring from few millimeters to 7.5 cm. The left ovary appeared normal and there are no deep lymph nodes or pelvic effusion (Figure 2). Laparotomy was performed under general anesthesia. It showed a large mass of 25/20 cm lobulated, with mixed solid and cystic components located in Douglas pouch and adherent to the posterior wall of the uterus. The uterus and adnexa appeared normal. After performing a cytological assay on peritoneal fluid, the mass was resected. Exploring the rest of the peritoneal cavity showed it to be free of abnormalities. The histopathological examination found a mass measuring 25/22 cm, with smooth and translucent external appearance (Figure 3). On section, the mass was midcystic and midsolid. Solid white areas were more or less firm and represented 70% of the surface. The microscopic examination depicted slight tumor proliferation with fusocellular cytonuclear atypies and low mitotic activity (1 mitosis/10 ×400 fields) (Figure 4). Additional immunohistochemical studies showed positivity for anti-CD34 and anti-CD99. Ki67 proliferation index is estimated to be <1% (Figure 4). Antibodies anti-AML, anti-desmin, anti-calretinin, anti-estrogen, and anti-progesterone were negative. The final diagnosis was an undifferentiated spindle cell tumor with immunohistochemical profile of a solitary fibrous tumor. Cytological examination of peritoneal fluid finds an inflammatory background rich in histiocytes without epithelial cells observed.

Once the diagnosis was established, a chest CT was performed in order to rule out a primary tumor and no abnormality was found.

The patient was followed by clinical and imaging examinations for the following four months.

## 3. Discussion

The term solitary fibrous tumor (SFT) is the current name to designate a tumor proliferation of spindle cells of mesenchymal origin. It was initially described in the pleura in 1931 by Klemperer and Rabin [1] and Biedrzycki [2]. This tumor is considered exclusively pleural. There has been a renewed interest since its description in multiple organs and locations, including soft tissue [3]. In the abdominopelvic space, many cases have been reported in the retroperitoneum, the pelvis, the peritoneal space, the presacral space, and the liver [4–6].

The pelvis is a rare localization of SFT [5]. It occurs most often in adults in their 50s, in both genders. In contrast, our patient was considerably younger at 32 years of age. This condition is often revealed by abdominal pain and sometimes by an insulin growth factor, usually type IGF II [7]. In a significant number of cases, it is asymptomatic and incidentally found on imaging. Our patient was referred from her family doctor for abdominopelvic mass associated with pelvic pain.

The ultrasound examination is carried out in a complementary first line; it allows for the specification of the characteristics of the mass with Doppler vascularity. Pelvic ultrasound of our patient depicted a right adnexal mass measuring 20 cm, which is heterogeneous, with tissular and cystic components and without intracystic or extracystic exophytic vegetations and without peritoneal fluid.

MRI usually shows an isosignal on T1-WI with a heterogeneous signal combining hyper-and hypointense zones in a patchy pattern on T2-WI, and a heterogeneous enhancement after gadolinium.

Macroscopically, they are whitish large masses of 10 cm (long axis), well delineated but not always encapsulated. In our observation, macroscopic examination found a white, smooth, and bumpy mass, measuring 25/22 cm, which is concordant with the literature.

Diagnosis of SFT can be difficult because of their unusual location and variability of pathological profile. Histological examination shows alternating areas of hypocellularity and hypercellularity along with thick bands of hyaline fibrosis. Cells can be round or fusiform. On immunohistochemistry, the majority of SFTs are positive for CD34 [2, 8, 9] and BCL-2 [9, 10]. They are generally negative for cytokeratins, smooth muscle actin, desmin, S-100 protein, and c-kit [9, 10]. These characters allows immunohistochemical differential diagnosis with other tumors [11–15] such as conjunctival angiomyofibroblastoma, cellular angiofibroma, giant cell angiofibroma, and fibroblastoma giant cell described by Hasegawa et al. [9, 10] and Fukunaga et al. [16].

The evolution of these SFTs is often slow and painless, except in the case of large tumors that may cause various cuts. Between 10 and 15% of SFTs are aggressive malignant seen with metastasis of the lung, liver, and bone. SFTs are often malignant. Hypercellular atypia shows moderate to marked cytonuclear with foci of tumor necrosis and numerous mitoses (more than four in ten fields at high magnification) [8, 10]. Our patient has none of these histologically worst criteria. The removal of the tumor remains the single most important factor influencing the evolution [5]. A staging imaging should be performed. It allows you to rule out a primary tumor (chest in order of frequency) which leads to pelvic secondary location and to perform a locoregional and general staging. Once the diagnosis is established, our patient underwent a chest CT which was normal.

Extrapleural SFTs are slowly progressing tumors with a low recurrence rate of 2 to 15%. The latter is related to the quality of the surgical resection and rare malignancy of extrapleural location [3, 6, 8]. Authors agree on the absolute necessity to achieve a wide surgical excision as complete as possible to avoid local recurrence. Because of their potential aggressiveness, it is recommended to perform an active followup each year.

A recent in vitro study showed that a solitary fibrous tumor with a mitotic index of 7/50 HPF benefits from chemotherapy, including 5-FU, adriamycin, mitomycin-C, and docetaxel. These treatments could benefit solitary fibrous tumors which are more aggressive, recurrent, unresectable, or with incomplete resection [17].

## 4. Conclusion

Pelvic solitary fibrous tumors are rare and most often benign as described in other localisations. However, malignant transformation is possible and can be correlated with some histological criteria. No consensus currently exists regarding these SFTs, but care must be maximized. No study has shown the effectiveness of additional treatment with chemotherapy or radiotherapy. Finally, a long-term followup is recommended for those patient who are at risk of relapsing.
